# Induction chemotherapy with paclitaxel and cisplatin to concurrent radiotherapy and weekly paclitaxel in the treatment of loco-regionally advanced, stage IV (M0), head and neck squamous cell carcinoma. Mature results of a prospective study

**DOI:** 10.1186/1748-717X-6-162

**Published:** 2011-11-22

**Authors:** Stefano Pergolizzi, Anna Santacaterina, Barbara Adamo, Tindara Franchina, Nerina Denaro, Pina Ferraro, Giusy RR Ricciardi, Nicola Settineri, Vincenzo Adamo

**Affiliations:** 1 Department of Radiological Science, University of Messina, Messina, Italy; 2 Operative Unit of Radiation Oncology, Azienda Ospedali Riuniti Papardo-Piemonte, Messina, Italy; 3 Department of Human Pathology, Division of Medical Oncology, University of Messina, Messina, Italy; 4 Operative Unit of Medical Physics, Azienda Ospedali Riuniti Papardo-Piemonte, Messina, Italy

**Keywords:** Chemoradiation, Induction Chemotherapy, Head and neck cancer, Radiotherapy

## Abstract

**Background:**

to evaluate activity and toxicity of a sequential treatment in advanced, non metastatic, mostly unresectable, head and neck squamous cell carcinoma.

**Methods:**

Patients with loco-regionally advanced or unresectable, head and neck cancer, were prospectively treated with 3 courses of induction chemotherapy followed by concurrent chemoradiation. Induction chemotherapy consisted of paclitaxel 175 mg/m2 day 1 and cisplatin 75 mg/m2 day 2, given every 3 weeks, to a total of three courses. Curative radiotherapy started 4 weeks after the last cycle of chemotherapy with the goal of delivering a total dose ≥ 66 Gy. During RT weekly paclitaxel (40 mg/m2) was administered.

**Results:**

The trial accrued 43 patients from January 1999 to December 2002. All patients received 3 courses of induction chemotherapy and the planned dose of radiotherapy. Thirty-eight patients were able to tolerate weekly paclitaxel during irradiation at least for 4 courses. After induction therapy there were 32 overall responses, 74.4% (23 partial and 9 complete); at completion of concomitant treatment overall responses were 42, 97.7% (20 partial and 22 complete). Median time to treatment failure was 20 months and the disease progression rate at 3 and 5 years was 33% and 23%, respectively. The median overall survival time was 24 months and 3 and 5 years overall survival rates were 37% and 26%, respectively. The major toxicity was mucositis.

**Conclusions:**

This combined treatment was found to be feasible and active in advanced or unresectable, head and neck squamous cell carcinoma patients. Long-term results observed in this trial encourage to consider this approach in further investigation using newer radiation delivering technique and new molecularly agents.

## Introduction

Advanced head and neck squamous cell carcinoma (HNSCC) has a poor prognosis. In fact, using surgery or radiation therapy, which have been the standard modalities used to treat this kind of disease, 5-year survival rates between 30 and 40% in locally advanced disease have been observed [1]. The MACH-NC [2] group showed that chemotherapy improved survival, 4% at 5 years, in advanced HNSCC with a higher benefit, 8%, using chemotherapy concomitantly to radiotherapy.

Induction chemotherapy to curative radiotherapy has not demonstrated to ameliorate clinical results with respect to concomitant chemo-radiation. However, a frequent finding from the induction studies was a lower incidence of distant metastases [3-5]. Using aggressive concurrent radio-chemotherapy regimens an improvement in loco-regional control has been observed, and the need to ameliorate the control of distant failures has become of interest [6].

The rationale in the use of an hybrid therapy with induction chemotherapy to concomitant radiotherapy and chemotherapy [7-9] is the purpose to ameliorate both distant and regional results.

Newer chemotherapeutic agents, such as paclitaxel, have been successfully integrated both in recurrent and metastatic HNSCC [10-12]; besides weekly paclitaxel can be safely combined with conventionally fractionated and hyperfractionated radiation [13,14].

In a dose-finding study using three courses of induction cisplatin/paclitaxel doublet to concomitant radiotherapy with paclitaxel we reported that the weekly paclitaxel MTD was 40 mg/m2 [15]. We adopted this dose level to start in 1999 a prospective study in advanced, unresectable HNSCC.

This prospective study was designed to determine activity and toxicity of this regimen; as secondary endpoints, we evaluated time-to-progression and overall survival with a median follow-up time of 9.25 years.

## Patients and methods

### Patient selection

Between January 1999 and December 2002, 43 patients were enrolled in this trial. In this study we present the treatment results after a minimum follow-up time greater than 8 years. The main eligibility criteria for this study were a histologically confirmed diagnosis of HNSCC, advanced or surgically unresectable, stage IV (M0) tumour as determined by the American Joint Committee on Cancer (AJCC) TNM staging system [16]. Patients with nasopharynx carcinoma, paranasal sinus cancer, and unknown primary site tumour were excluded as well as patients with prior or active concurrent malignancy.

Patients were required to have a measurable disease and an ECOG performance score ≤ 2. Additional eligibility criteria included age ≥ 18 years, life expectancy ≥ 6 months, adequate renal, hepatic and bone marrow function (haemoglobin ≥ 10.5 g/dL, granulocyte count ≥ 2.0 × 109/L, platelet count ≥ 100 × 109/L, serum bilirubin ≤ 1.25 × normal, serum albumin ≥ 35 g/dL, serum creatinine ≤ 120_mol/L (≤ 1.5 mg/dL), and creatinine clearance ≥ 50 mL/min). Patients were evaluated and treatment recommendations were made at a multidisciplinary University Hospital that included head and neck surgeons, radiation oncologists and medical oncologists. The study was reviewed by the University of Messina Institutional Review Board for Plans of Research. All patients went through an informed consent process prior to entry on the study.

### Clinical assessment

Patients evaluation included medical history and physical examination; complete blood count and blood tests; panendoscopy with multiple biopsies; standard chest X-ray; liver ultrasound; both CT and MR were requested by protocol either to confirm or to exclude the feasibility of a surgical approach. Other investigations were performed in the presence of clinically suspicious signs.

### Treatment plan

#### - Induction Chemotherapy

Patients were submitted to a course of paclitaxel 175 mg/m2 day 1 and cisplatin 75 mg/m2 day 2 given every 3 weeks for a total of three courses; paclitaxel was administered as a 3-hour infusion with a standard premedication including dexamethasone, oral ondansetron, clorfenamine and ranitidine. The prophylactic use of colony-stimulating factors and/or erythropoietin was not permitted.

#### - Radiotherapy with weekly paclitaxel

Curative irradiation started 4-5 weeks after the last cycle of chemotherapy with the goal of delivering a total dose of 66-70 Gy (2 Gy daily fraction, 5 days per week) on primary tumour.

For treatment planning, a CT scan of the head-neck (from base of the skull to inferior edge of clavicula) was mandatory using a slice thickness of 5-8 mm while the patient was immobilized in a supine position on a head-neck board.

The preinduction gross tumor volume was reconstructed for treatment planning and a maximum dose of 42 Gy was delivered to the spinal cord; our technique treatment details have been previously reported [17]. Briefly, we used two lateral fields, with customized blocks, to treat the upper neck and the primitive tumour and an anterior field to treat both the lower neck and supraclavicular nodes; a "shrinking field" technique was employed to spare the spinal cord and the posterior neck was treated using electrons. 50 Gy were delivered to clinically uninvolved nodes and 64-66 Gy to positive nodes.

All patients received supportive care during radiotherapy, including dietary measures, local antiseptics, prophylactic use of cortisone and fluconazole.

In order to maintain body weight, total enteral nutrition therapy was performed using nasogastric tube or PEG in patients with grade 3+ mucositis; finally, we chose to treat with trans-dermal fentanyl all patients who presented grade 3-4 mucositis with dysphagia.

During radiotherapy weekly paclitaxel, 40 mg/m2, was administered for 6 courses if feasible, using standard premedication.

#### - Surgery

Whenever feasible surgery was recommended to all patients with post treatment clinical N1-2 who had a response greater than 80% on primary tumour site.

### Toxicity assessments

During treatment we monitored patients for signs and symptoms of toxicity. Toxicities were evaluated by physical examination and by laboratory blood cell counts. Toxicity was graded according to the common toxicity criteria version 2.0 and the grade reported was the worst observed grade of each toxicity that was experienced by a patient.

### Definition of clinical response and Follow-up

Patients were evaluated by CT scan 4 weeks after induction chemotherapy, and by chest X-ray and CT scan at 6 weeks after chemoradiation treatment. A clinical complete remission (CR) was defined as a complete disappearance of all detectable disease for 4 weeks or more; partial remission (PR) was assessed as a greater than 50% decrease in the sum of the products of the two longest perpendicular diameters of all measurable lesions persisting for at least 4 weeks; no change (NC), or stable disease, when a less than 50% response or a progression of the lesion/s < 25% were detected; progressive disease (PD) was considered to be a volumetric increase in tumor and/or node/s by > 25% and/or the occurrence of "new lesions".

All patients were to be followed for the duration of their life, every 4 months in the first and second year, and yearly afterwards; the date and site of first local-regional or distant failure were registered. In case of failure, the choice of treatment was made on an individual basis.

### Statistical analysis

Because of the mono-institutional study, we decided to accrue prospectively a consecutive series of patients during a pre-defined 4 years time.

The primary end point was toxicity and response rate. As secondary endpoints, we also evaluated the progression-free survival (PFS) and overall survival (OS). PFS is defined as time from registration to tumour recurrence. OS was defined as time from the date of registration to date of death. Patients who died without documented recurrence were excluded at the date of death.

Enrollment was started in January 1999 and closed in December 2002, after 43 patients had been registered. Results were analyzed on April 2011, after a median follow-up of 111 months.

Statistical analysis of overall survival and time-to progression disease were performed using a one-sided log-rank of Kaplan-Meier survival estimates [18].

## Results

### Patient characteristics

Table 1 shows both patients' and tumours' characteristics. Median age was 60 years (range 29-74 years). There were 38 male and 5 female. Larynx (32%) and Oropharynx (28%) were the most common primary tumour site. Thirty-five tumours were moderately-poor differentiated and there were twenty-one patients with a T4 stage. The presence of Human Papillloma Virus (HPV) and p16 status was not evaluated. Forty patients had nodal involvement, 21 N2 and 9 N3. IVA stage was assigned in 34 cases. All patients were assessable for toxicity and response.

**Table 1:** Baseline Demographic and Clinical Characteristics of the study Population (N.43)

***Sex***	
*Male*	*38*
*Female*	*5*
	
***Age, years***	
*Median*	*60*
*Range*	*29-74*
	
***Tumor Site***	
*Hypopharynx*	*7*
*Oral cavity*	*10*
*Oropharynx*	*12*
*Larynx*	*14*
	
***Tumor Grading***	
*G1*	*8*
*G2*	*19*
*G3*	*16*
	
***Tumor Stage***	
*T1*	*1*
*T2*	*7*
*T3*	*14*
*T4*	*21*
	
***Nodal stage***	
*N0*	*3*
*N1*	*12*
*N2a-c*	*20*
*N3*	*8*
	
***TNM Stage***	
*T1 N3*	*1*
*T2 N2b*	*2*
*T2 N2c*	*3*
*T2 N3*	*2*
*T3 N1*	*1*
*T3 N2a*	*3*
*T3 N2b*	*2*
*T3 N2c*	*4*
*T3 N3*	*4*
*T4 N0*	*3*
*T4 N1*	*11*
*T4 N2a*	*1*
*T4 N2b*	*1*
*T4 N2c*	*4*
*T4 N3*	*1*
*Stage III*	*1*
*Stage IVA*	*34*
*Stage IVB*	*8*

### Treatment compliance

All patients completed the planned induction chemotherapy for a total of 129 courses; radiation therapy was fully delivered in 43 patients and 33 (77%) patients were able to tolerate concurrent paclitaxel for at least 5 courses. Interruptions of irradiation due to toxicities have been necessary in 4 patients; the break time was, respectively, of 3, 4, 6, and 8 days. One patient refused weekly paclitaxel after the first course. A total of 216 paclitaxel courses were administered during study time. Table 2 details the delivered courses of chemotherapy. One-hundred percent of patients received full planned dose radiotherapy with a median dose of 70 Gy (range 66-70 Gy). There were no toxic deaths.

**Table 2:** Courses of chemotherapy delivered in 43 patients.

	1	2	3	4	5	6	7	Total
Induction chemotherapy			43					129
Weekly paclitaxel	1	3	1	5	16	12	5	216

### Treatment-related toxicites

All patients showed epithelitis and alopecia and these did not exceed grade 3. Mucositis occurred in 43 patients; 32 patients presented grade 2 mucositis and 11 grade 3-4. A patients reported a grade 4 nausea/vomiting. Grade 3 neutropenia and thrombocytopenia was respectively observed in 8 and 2 patients. Peripheral neuropathy occurred in 5 patients and it had a grade 3 in 2 patients. The toxicities are listed in Table 3.

**Table 3:** Cumulative toxicities in 43 patients

	Pt number	G1	G2	G3	G4
Skin	43	3	37	3	
Neutropenia	21	5	8	8	
Alopecia	43		43		
Mucositis	43		32	6	5
Nausea/vomiting	6	3	2		1
Allergy	2	1	1		
Diarrhea	3	1	2		
Anemia	10	7	3		
Piastinopenia	15	11	2	2	
Peripheral Neuropathy	5	1	2	2	

### Response to treatment

After three cycles of induction chemotherapy, nine patients (20.9%) had a complete tumour response and 23 patients (53.5%) had a partial response. Stable disease was observed in ten patients (23.3%). A patient experienced disease progression during induction chemotherapy. Initial evaluation after chemoradiotherapy showed that overall responses were complete in 51.2% (22 patients), partial in 46.5% (20 patients), and stable in a patient. 12 patients required post-treatment neck dissections and in 3 cases there was no pathologic evidence of persistent disease. Objective responses are shown in Table 4.

**Table 4:** Overall response to induction treatment and concurrent radiochemotherapy

Response	**N**.	(%)
Induction Treatment		
Complete	9	(20.9)
Partial	23	(53.5)
Overall response	32	(74.4)
Stable Disease	10	(23.3)
Progression Disease	1	(2.3)
		
After concurrent radiochemotherapy		
Complete	22	(51.2)
Partial	20	(46.5)
Overall response	42	(97.7)
Stable Disease	1	(2.3)
Progression Disease	0	(0)

Tumour control, patterns of failure. Time to progression and survival analysis.

**Figure 1:**
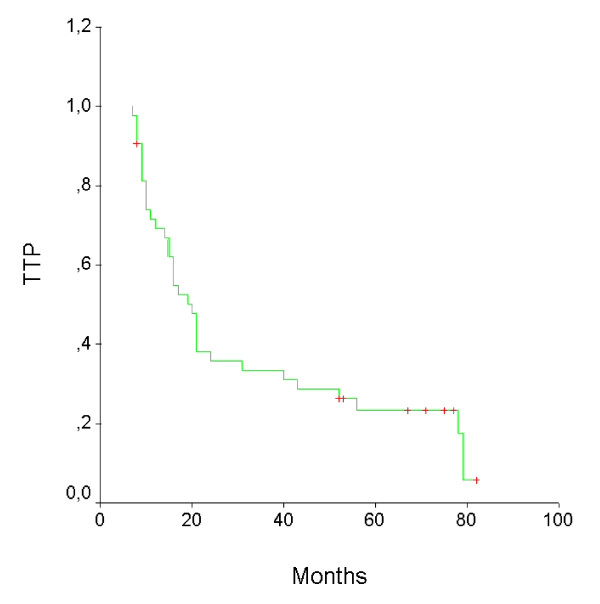
**Time To Progression curve for 43 patients**.

**Figure 2:**
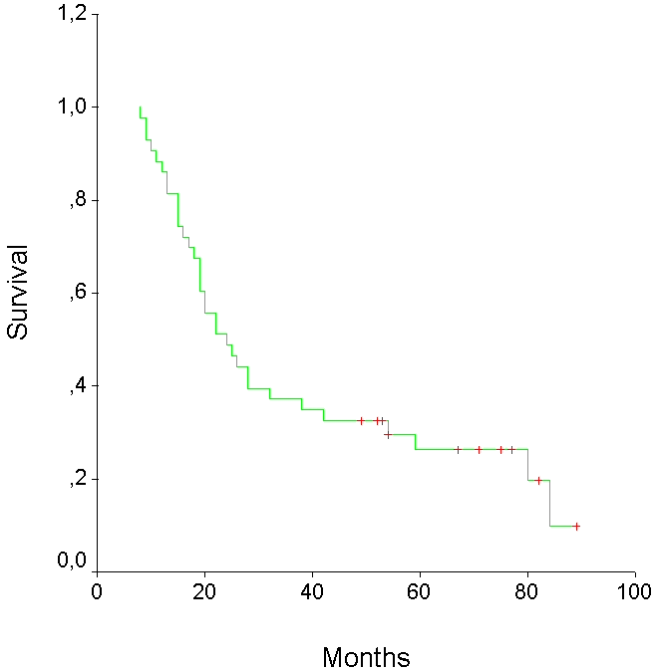
**Overall survival curve for 43 patients**.

To date, 36 patients have progressed: ten patients at primary site, six in regional nodes, fourteen with distant metastases, and six with primary site or regional nodes, and distant metastases (Table 5). With a median follow-up of 111 months, median time to treatment failure was 20 months and the disease progression rate at 3 and 5 years was 33 and 23%, respectively. The median overall survival time was 24 months and 3 and 5 years overall survival rates were 37% and 26%, respectively. The survival curves are shown in Figures 1 and 2.

**Table 5:** Site/s of first relapse in 43 patients

Local/regional	Local/regional and distant	Distant
16 (10 Primary tumour 6 Regional nodes)	6	14

### Long-term toxicities

In 10 patients severe salivary glands impairment was reported; in a case severe hearing loss appeared 4 years after the end of therapies; 3 patients had feeding tube dependence for 12, 14 and 22 months respectively.

## Discussion

Induction chemotherapy to curative chemo-radiation in treating HNSCC has been studied in several trials. At present, no schedule can be considered standard care in this setting and the therapeutic benefit of either induction or consolidation full-dose chemotherapy in conjunction with concurrent chemoradiotherapy is not yet clearly established especially in the era of HPV associated disease. Moreover, a conclusive statement on any improvement in survival over that seen with concurrent chemotherapy and radiotherapy cannot be made at the current time [19].

However, comparing two-drugs versus three-drugs regimen in randomized studies, some Authors report better results when 3-drugs combination are employed [20-22].

This study is a prospective trial testing the hypothesis that the use of paclitaxel both in neo-adjuvant regimen and concurrent with irradiation, is feasible with acceptable toxicities in unresectable or local-regionally advanced HNSCC.

The regimen used in our study is somewhat unique in that it omits 5-FU as part of the induction and does not contain a platinum with concurrent radiation; besides it employs paclitaxel because at the time of study design docetaxel was not available. It reflects a regimen which is similar to that used by physicians in the community and gives a more accurate reflection of the toxicity and activity than other reports.

The distinctive features of our study are the combination of a two-drug induction schedules followed by chemoradiation with a single drug in order to minimize the side effects. At the time of study design the standard chemotherapy regimen using doublets was cisplatin/5 fluorouracil but both prospective [10,11] and randomized studies demonstrated that there was neither a difference in overall survival nor in response rate between cisplatin/5 fluorouracil and cisplatin/paclitaxel [12] in recurrent or metastatic HNSCC.

Using our regimen we observed manageable toxicities during induction chemotherapy; in fact 43/43 patients were able to tolerate the treatment. It is noteworthy that in the concurrent phase of therapy, all patients completed the planned radiotherapy and in 38/43 (88.4%) of cases at least 4 weekly administration of paclitaxel were performed. This in spite in our study the prophylactic use of colony-stimulating factors and/or erythropoietin was not permitted. Probably, the use of enteral nutrition and opioid in patients who had G3+ toxicities has an important role; since there is no standard supportive care during radiotherapy, this hypothesis must be considered as conjectural.

Moreover, the radiation delivering techniques used for this trial up-to-date have to be considered out of standard; in fact we do not applied 3D conformal radiation therapy or intensity-modulated radiotherapy (IMRT). Compared with conventional techniques of irradiation, intensity-modulated radiation therapy indeed allows better sparing of healthy tissues; the subsequent reduction in radiation induced toxicities may help to ameliorate the patient' compliance to intensive concomitant chemoradiotherapy regimens.

After induction therapy overall response rate was 74.4% (32 patients) with complete and partial response rates of 20.9% and 53.5% respectively. These results overlap with the high response rates observed in other studies in which paclitaxel is used in induction combinations [7,23]. Besides, an high overall response rate (97.7%) has been observed also at the end of concurrent phase of treatment. In the ECOG2399 trial (two courses of induction paclitaxel and carboplatin followed by weekly paclitaxel at 30 mg/mq concurrent with irradiation), Cmelak et al [24] reported 68% overall response rate (38% complete and 30% partial) at initial evaluation after chemoradiotherapy. Probably, the better response rate observed in our patients may be due to the use of three courses of induction chemotherapy and 40 mg/mq weekly paclitaxel (our study).

The inclusion of stage IV (M0) patients only is an important feature to demonstrate the real value of chemoradiation in this setting, as reflected in the 26% overall survival at 5 years. Our group of patients were homogeneous with stage IV disease, but most studies included patients with stage III and IV disease. This difference should be taken into account when comparing results. In fact in this study 100% of the patients had stage IV disease, mostly had unresectable tumors, and the majority of the patients did not have laryngeal primary tumors.

In summary, our study seems to indicate that induction chemotherapy with paclitaxel and cisplatinum followed by concurrent paclitaxel and radiotherapy is feasible and has activity with a reasonably good response rate comparable with those evident in the literature with standard concurrent chemo-radiotherapy. Our regimen is manageable with an acceptable toxicity profile, and it is surely not life-threating; these findings are important because the toxicity of chemoradiation, even in the absence of any prior treatment, can be considerable and its tolerability is poor in a significant number of patients [25]. We observed also a low incidence of severe late toxicities, in fact prolonged use of feeding tube was necessary only in three case and for a period inferior to two years. The high incidence of severe late damage to salivary glands (10/43 patients) observed in our patients probably is due to the techniques employed to deliver radiotherapy in our study. To overcome this limitation we are exploring induction chemotherapy followed by concurrent chemo-irradiation using IMRT.

Moreover, considering the safety of our schema, this sequential therapy could be considered an appropriate platforms upon which both the modern techniques delivering radiation and new molecularly targeted agents could be tested.

## Competing interests

The authors declare that they have no competing interests.

## Authors' contributions

SP designed the study, analysed the data and prepared the manuscript. BA, SA, TF, ND, PF and GR extracted, and sorted the data; participated in the treatment of the patient cohort described, and assisted with preparation of the manuscript. NS conducted all statistical analyses. VA designed the study, analysed the data and prepared the manuscript. All authors read and approved the final manuscript.
